# Detection of Porcine Circovirus (PCV) Using CRISPR-Cas12a/13a Coupled with Isothermal Amplification

**DOI:** 10.3390/v16101548

**Published:** 2024-09-30

**Authors:** Huijuan Wang, Gang Zhou, Huiming Liu, Ruqun Peng, Tingli Sun, Sujuan Li, Mingjie Chen, Yingsi Wang, Qingshan Shi, Xiaobao Xie

**Affiliations:** Guangdong Provincial Key Laboratory of Microbial Culture Collection and Application, State Key Laboratory of Applied Microbiology Southern China, Institute of Microbiology, Guangdong Academy of Sciences, Guangzhou 510070, China; wanghj@gdim.cn (H.W.); zgbees@gdim.cn (G.Z.); liuhuiming@gdim.cn (H.L.); pengruqun@gdim.cn (R.P.); suntingli@gdim.cn (T.S.); lisujuan@gdim.cn (S.L.); chenmj@gdim.cn (M.C.); wongvincy@163.com (Y.W.); shiqingshan@hotmail.com (Q.S.)

**Keywords:** porcine circovirus, CRISPR-Cas12a, CRISPR-Cas13a, isothermal amplification, rapid detection

## Abstract

The impact of porcine circovirus (PCV) on the worldwide pig industry is profound, leading to notable economic losses. Early and prompt identification of PCV is essential in managing and controlling this disease effectively. A range of detection techniques for PCV have been developed and primarily divided into two categories focusing on nucleic acid or serum antibody identification. The methodologies encompass conventional polymerase chain reaction (PCR), real-time fluorescence quantitative PCR (qPCR), fluorescence in situ hybridization (FISH), loop-mediated isothermal amplification (LAMP), immunofluorescence assay (IFA), immunohistochemistry (IHC), and enzyme-linked immunosorbent assay (ELISA). Despite their efficacy, these techniques are often impeded by the necessity for substantial investment in equipment, specialized knowledge, and intricate procedural steps, which complicate their application in real-time field detections. To surmount these challenges, a sensitive, rapid, and specific PCV detection method using Clustered Regularly Interspaced Short Palindromic Repeats (CRISPR)-Cas12a/13a coupled with isothermal amplification, such as enzymatic recombinase amplification (ERA), recombinase polymerase amplification (RPA), and loop-mediated isothermal amplification (LAMP), has been developed. This novel method has undergone meticulous optimization for detecting PCV types 2, 3, and 4, boasting a remarkable sensitivity to identify a single copy per microliter. The specificity of this technique is exemplary, with no observable interaction with other porcine viruses such as PEDV, PRRSV, PRV, and CSFV. Its reliability has been validated with clinical samples, where it produced a perfect alignment with qPCR findings, showcasing a 100% coincidence rate. The elegance of merging CRISPR-Cas technology with isothermal amplification assays lies in its on-site testing without the need for expensive tools or trained personnel, rendering it exceptionally suitable for on-site applications, especially in resource-constrained swine farming environments. This review assesses and compares the process and characteristics inherent in the utilization of ERA/LAMP/RPA-CRISPR-Cas12a/Cas13a methodologies for the detection of PCV, providing critical insights into their practicality and effectiveness.

## 1. Introduction

The porcine circovirus (PCV) belongs to the *Circovirus* genus of the *Circoviridae* family and has a circular single-stranded DNA (ssDNA) of roughly 1.7–2.0 kb in length, making it one of the smallest animal viruses ever discovered [1,2]. PCV was first discovered in 1974, and the taxonomy of PCV encompasses four distinct species up to now: PCV1, PCV2, PCV3, and the recently characterized PCV4 [3,4]. PCV1, initially isolated from porcine kidney cells, has been subsequently recognized as non-pathogenic in pigs [5,6]. Nevertheless, PCV2, which was first discovered in Canada in 1991, targets porcine lymphoid tissue and causes various diseases in pigs, such as diarrhea, central nervous system disorders, and symptomatic disease [7,8,9], all of which have been associated with serious economic detriment to the swine industry [10]. The emergence of PCV3 in the United States in 2016 via metagenomic sequencing has brought additional challenges, as afflicted piglets exhibit symptoms similar to porcine dermatitis and nephrotic syndrome (PDNS), reproductive dysfunction, and multisystemic inflammation [11,12,13]. Identified in 2019, PCV4 has a notable presence in Chinese pigs, with prevalence rates of 3.3–25.4% across various provinces [14,15,16]. Though its pathogenic mechanisms are not fully elucidated, PCV4 has been associated with a diverse array of clinical symptoms, including postweaning multisystemic wasting syndrome (PMWS), PDNS, and respiratory and neurological symptoms, as well as reproductive dysfunction [17]. In addition, PCV4 shows the highest genomic identity to mink circovirus (66.9%) and identities to the other PCV genomes ranging from 43.2% to 51.5% (PCV1: 50.3%; PCV2: 51.5%; PCV3: 43.2%) [14].

PCVs, particularly PCV2 and PCV3, bring a severe economic burden to the global swine industry [3,18]. The virus is linked to a variety of diseases, including PDNS, PMWS, and reproductive failure, all of which cause increased feed costs, reduced growth performance, high mortality rates, and higher veterinary expenses, resulting in substantial financial losses for pig producers [19,20]. Except for PCV1, early detection and surveillance of PCV2, PCV3, and PCV4 are critical for related infectious disease prevention and control measures construction. To date, a plethora of diagnostic modalities have been documented for PCV detection, primarily bifurcated into assays targeting nucleic acid identification or serum antibody presence [21,22]. The spectrum of these diagnostic techniques encompasses a range of methodologies, including the conventional polymerase chain reaction (PCR) [23,24], real-time fluorescence quantitative PCR (qPCR) [25,26], droplet digital polymerase chain reaction (ddPCR) [27,28], fluorescence in situ hybridization (FISH) [29], colorimetric isothermal multiple-self-matching-initiated amplification (IMSA) [30], immunofluorescence assay (IFA) [31], immunohistochemistry (IHC) [32,33], gold nanoparticle-based immunochromatographic strip (NBIS) [34], and enzyme-linked immunosorbent assay (ELISA) [35,36,37]. Despite being widely used in the detection of PCVs, these diagnostic approaches involve complex procedures, specialized instruments, and professional personnel. Similarly, because of their low sensitivity and vulnerability to contamination, PCV antigen detection techniques like ELISA and IFA are not as trustworthy. In addition, high costs, lengthy development times, and the inability to conduct on-site testing render all the above methods unsuitable for widespread development and usage in remote and underdeveloped nations. These issues virtually eliminate the possibility of real-time detection in the field.

Hence, for PCV point-of-care detection, a highly sensitive, quick, and visible diagnostic technique is required. Numerous researchers have investigated a few PCV detection strategies in conjunction with isothermal amplification techniques since the discovery of CRISPR. This review provided direction for the development of more sophisticated PCV detection technology by summarizing and contrasting the published CRISPR-based PCV detection methods.

## 2. CRISPR-Cas

In bacteria and archaea, CRISPR-Cas systems act as adaptive immune systems, protecting against external genetic materials [38]. The CRISPR arrays, containing the history of past foreign nucleic acid attacks, incorporate new spacers during infections [39,40]. Subsequently, prokaryotes develop adaptive immunity by forming RNA-guided endonucleases that recognize and target foreign nucleic acids [41]. There are two main classes of CRISPR-Cas systems: Class 1 and 2 systems employ multiple and a single Cas protein, respectively, in their CRISPR ribonucleoprotein effector nucleases [42]. Class 1 and 2 CRISPR-Cas systems make up around 90% and 10% of all known CRISPR-Cas loci, respectively [43]. CRISPR-Cas systems form a ribonucleoprotein complex with CRISPR RNA (crRNA) and a Cas protein, targeting specific DNA sequences through complementarity with crRNA after identifying the Protospacer Adjacent Motif (PAM) sequence [41,44,45]. Specifically, the next generation of molecular rapid diagnosis technology is thought to be the developed detection methods based on the trans-cleavage activities of Cas12a and Cas13a proteins [46,47]. Meanwhile, isothermal techniques were developed to amply target nucleic acids by requiring only one incubation at a single temperature, which eliminates the need for additional sophisticated temperature control equipment [21]. These techniques include isothermal enzymatic recombinase amplification (ERA) [13], recombinase polymerase amplification (RPA) [48,49,50], and loop-mediated isothermal amplification (LAMP) [51,52]. Therefore, to prevent and manage multiple PCVs, scientists have integrated ERA, RPA, and LAMP amplification technologies to create a variety of CRISPR/Cas system-based detection platforms, which exhibit excellent performance in terms of fast, highly sensitive, and precise viral detection [53].

### 2.1. CRISPR-Cas12a

Researchers at the Broad Institute of MIT and Harvard University initiated the discovery and characterization of the Cas12a system. They found a set of CRISPR nucleases in *Prevotella* and *Francisella* 1 bacteria that they named Cas12a (Cpf1), which correspond to the Class 2, type V CRISPR system [54]. Cas12a requires the PAM sequence “TTN/TTTN/TTTV” (N = A/T/C/G; V = A/C/G), and there are usually three homologs of the Cas12a nucleases: FnCas12a (from *Francisella novicida*), LbCas12a (from *Lachnospiraceae bacterium*), and AsCas12a (from *Acidaminococcus* sp.) [54], which are commonly used in plant genome editing technologies.

The two halves of the CRISPR-Cas12a system are a single crRNA and a protein/effector nuclease. FnCas12a, LbCas12a, and AsCas12a proteins exhibit comparable domain organizations and range in size from approximately 1300 to 1307 amino acids. A bi-lobed organization made up of an α-helical recognition lobe (REC) and a nuclease lobe (NUC) is shown by the Cas12a crystal structure [55,56]. The RuvC nuclease domain and three supplementary domains, PI, WED, and BH, make up the NUC lobe, whereas the two domains Hel-1 and Hel-2 compose the REC lobe. Cas12a’s RuvC endonuclease domain is separated into three discontinuous segments (RuvC I-III). Unlike Cas9 proteins, however, it does not include the second HNH endonuclease domain and processes its mature crRNA without using trans-activating CRISPR RNA (tracrRNA) [57,58].

In a previous study, Lei et al. aimed to develop a novel and efficient method of PCV2 detection by combining the advantage of LAMP, which does not require specific equipment, with the capability of the CRISPR-Cas12a system, which can cleave an ssDNA fluorophore quencher probe sensor (designed as LAPM-CRISPR) through the huLbCas12a collateral cleavage activity [59]. Briefly, the PCV2 viral DNA was extracted with a TIANamp kit, followed by an LAMP primer design targeting the *rep* gene’s conserved sequence. CRISPR-DT was utilized for three pairs of crRNA designs, with huLbCas12a expression and purification facilitated by the pET-28a vector. An FAM-BHQ1-labeled ssDNA was integrated for UV visualization. The LAMP-CRISPR-Cas12a system was optimized for performance, with fluorescence measured by ABI QuantStudio 5. Sensitivity and specificity were evaluated against various PCVs, with qPCR as the benchmark for clinical sample assessment. The results showed that the crRNA2&3 targeting group exhibited high fluorescence with a low background, indicating strong UV-induced fluorescence. Optimal huLbCas12a protein and crRNA concentrations were 250:250 nM, achieving peak fluorescence intensity and facilitating binding. The detection limit was 1.0 copies/µL, consistent with ABI QuantStudio 5 analysis. This method accurately distinguished PCV2 from other viruses based on fluorescence intensity differences, demonstrating sensitivity and specificity. Reliability was confirmed by comparing LAMP-CRISPR with qPCR in 30 clinical samples, showing complete consistency [59].

Similarly, LAMP-CRISPR/Cas12a was also explored and conducted [60], whose basic methods are comparable to those of Lei et al. [59]. Meanwhile, there are some differences between these two studies. Firstly, the PCV2 ORF2 gene was identified as the target. Secondly, four LAMP- and five CRISPR-specific primers were designed. Thirdly, the results demonstrated that the minimum detection threshold of this approach was nearly equal to one copy of plasmid DNA. Finally, visual monitoring of reaction products under blue light was made possible by the LAMP-CRISPR/Cas12a results [60].

PCV3 was also diagnosed using CRISPR-Cas12a in conjunction with ERA nucleic acid amplification [13]. Firstly, the detection target is the *rep* gene of PCV3. Secondly, primers for the ERA reaction were designed as a single pair. Thirdly, a probe sensor labeled with an ssDNA fluorophore quencher (FAM-N6-BHQ1) was employed. Fourthly, the results can be ascertained in the presence of LED blue light, with the lowest limit of detection (LOD) at 7 copies for visual observation [13].

In addition to the use of ssDNA fluorophore quencher-labeled probe sensors, such as FAM-N6-BHQ1, CRISPR-based PCV detection also employed hemin/G-quadruplex DNase activity to develop glucose oxidation and biosensors [61]. In that study, a short G-rich DNA sequence PW17, which is frequently utilized to construct G-quadruplex monomers, was synthesized, and Cas12a’s trans-cleavage of ssDNA post-target dsDNA cleavage was utilized. Target sequences amplified by RPA were cut by Cas12a and crRNA, activating Cas12a’s trans-cleavage. Activated Cas12a cut G-quadruplex ssDNA, preventing spatial structure formation and oxidase activity. Without a target, G-quadruplex maintained oxidase activity, combining with hemin to catalyze TMB into blue. Positive reactions were colorless, negative blue. RPA amplified clinical samples in vitro, with CRISPR-Cas12a and G-quadruplex detecting a minimum of 10^3^ copies [61].

### 2.2. CRISPR-Cas13a

A type VI RNA-guided RNase, Cas13a (also known as C2c2), has two conserved Higher Eukaryotes and Prokaryotes Nucleotide-binding (HEPN) domains [62]. The crRNA recognition (REC) and the nuclease (NUC) lobes are the two lobes that make up Cas13a. The NUC lobe contains the HEPN1, HEPN2, and Helical-2 domains as well as a linker between two HEPN domains, whereas the REC lobe is made up of the Helical-1 domain and the N-terminal domain (NTD) [63]. A 3′-spacer and a 5′-handle, which are further subdivided into 5′-flank, 5′-stem, loop, 3′-stem, and 3′-flank, are present in mature crRNA [64]. Pre-crRNA is separately processed by Cas13a into mature crRNA, which it then combines with its crRNA to create the surveillance complex that cleaves both the target and surrounding RNAs upon recognition of the foreign target RNA [64]. By utilizing its distinctive collateral activity, *Leptotrichia wadei* (Lwa)’s LwaCas13a, a CRISPR-Cas13a variation, has been rapidly produced for nucleic acid-based diagnostics [65]. CRISPR-LwaCas13a can be used to detect targeted RNAs by using a fluorophore–quencher pair connected by an ssRNA that fluoresces following cleavage by active LwaCas13 [66,67].

In 2019, PCV4 emerged in Hunan, China, and has since been detected in various regions, posing a significant risk to the swine industry [68,69,70,71]. To address this, Jieru Wang and colleagues engineered a CRISPR-Cas13a-based lateral flow strip assay for the rapid and sensitive detection of PCV4. The assay leverages a synthesized segment of the PCV4-Cap gene to design specific crRNAs and employs an FAM-N6-BIO-labeled probe for detection. Following the synthesis of crRNA and the probe, the team validated the method using 15 clinical samples, achieving high accuracy and demonstrating the ability to identify PCV4 from a single-copy template in under 1.5 h, showcasing its potential for on-site application [72]. This innovative approach ensures both sensitivity and specificity, crucial for early PCV4 detection and infection control within the swine industry [72].

## 3. Comprehensive Comparison of Their Pros and Cons

### 3.1. Primers and crRNA

Isothermal amplification reaction primers must be designed for all the studies mentioned above. Generally, three pairs of primers minimum are needed for LAMP. Four [60] or six pairs [59] of LAMP primers were created in the detection of PCV2. In contrast, one pair of primers is sufficient for isothermal amplification for ERA [13] or RPA [61,72]. However, the cost of PCV detection increased because a commercial kit for isothermal amplification is required for an ERA or RPA reaction. Meanwhile, crRNA may have an impact on the detection efficiency. Therefore, the authors designed and employed several crRNAs for PCV detection. However, only one crRNA was used in the visual and label-free PCV2 detection by CRISPR-Cas12a in conjunction with G-quadruplex [61]. Consequently, the LOD of this investigation was 10^3^ copies/µL, which is apparently greater than the other studies. Therefore, we strongly recommend designing additional crRNAs and optimizing them. Furthermore, the crRNA2&3 double targeting group displayed a comparatively low background fluorescence value despite having a higher fluorescence value, indicating that they may be able to induce strong fluorescence when exposed to UV radiation [59].

### 3.2. Commercial Kits

Typically, multiple commercial kits were needed for the entire PCV detection process based on EPA/ERA/LAMP-Crispr-Cas12a/13a. For instance, a viral DNA/RNA extraction kit is needed to extract the PCV genome [13,59,72]. A basic ERA/EPA nucleic acid amplification kit or LAMP Master Mix is required for isothermal amplification [13,59,60,61,72]. Use of commercial reagent kits, if available, is strongly advised to increase testing accuracy and convenience. The cost of testing is increased when commercial test kits are used, but the accuracy and convenience of testing are significantly enhanced, particularly regarding accuracy.

### 3.3. Cas Proteins

Within the context of the literature discussed (Table 1), Cas12a or Cas13a was utilized. However, just one study independently expressed and purified the Cas12a protein by themselves [59]. The commercial Cas12a protein, purchased from New England Biolabs (NEB) (Ipswich, MA, USA), was used in other papers [13,60,61]. LwaCas13a was obtained from Magiltd, Hefei, China [72]. For convenience, we strongly recommend using commercially supplied Cas proteins because it takes a lot of effort and time to express and purify the Cas12a/13a protein. Meanwhile, a skilled operator is required for the creation of the expression plasmid, transformation, induced expression, protein collection, and purification. Furthermore, prior to conducting the official research, you also need to confirm and evaluate the expressed Cas protein’s cleavage activity and efficiency, while for commercial Cas proteins, you can use it directly. In addition, the PAM of Cas12a is TTTN, where N stands for any nucleotides. However, there is no PAM needed for Cas13a. As a result, the criteria for crRNA designation for Cas13a are somewhat relaxed. But in Cas13a detection, the viral DNA targets of PCV2, PCV3, and PCV4 have been reverse-transcribed into RNA. Consequently, in contrast to Cas12a detection, it introduced an extra step for Cas13a detection.

### 3.4. Fluorescence Probes

An appropriate probe is crucial for a detection technique. Two different types of probes have been employed in the ERA/LAMP/RPA-CRISPR-Cas12a/13a to identify PCV: FAM-BHQ1/BIO [59,60,61,72] and G-quadruplex [13]. If they work with the related system well, these probes are not inherently good or bad. More importantly, UV light has the potential to trigger fluorescence, which is visible to the naked eye. The results might be easily observed further if ERA/LAMP/RPA-CRISPR-Cas12a/13a is coupled with a lateral flow strip (LFD) [72]. But the cost of testing would go up. G-quadruplex, on the other hand, displayed the lowest LOD of 10^3^ copies [13]. It could therefore be further optimized.

### 3.5. Detection Times

**Figure 1:**
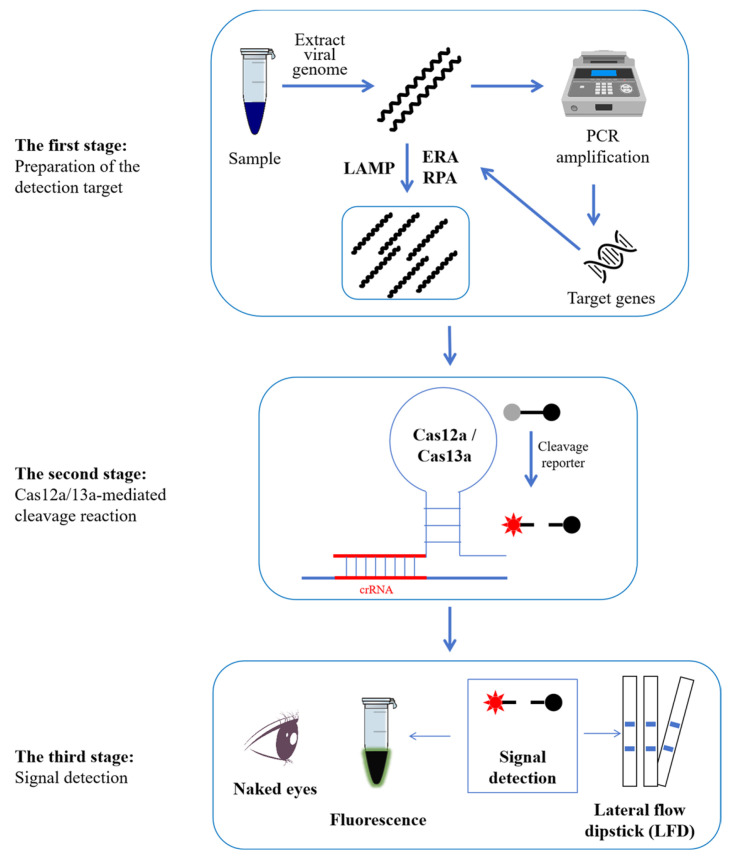
Schematic diagram of the workflow of PCV detection employing the ERA/LAMP/RPA-CRISPR-Cas12a/Cas13a methodology. The comprehensive process can be divided into three stages. In the first stage, the genomic DNA of PCVs was first extracted. And then, the detected targets were amplified using ERA, LAMP, or RPA. In the second stage, the Cas12a or Cas13a protein would locate the target genes with the guidance of crRNA, and then the collateral activity of Cas12a/13a would be induced to cut the probes. In the third stage, the fluorescence signal will be activated using fluorescent equipment and can be observed using the naked eye. If not, the results can be seen as distinct lines when an LFD is submerged in the reaction tubes.

Another crucial component in site detection is reaction time. In the studies mentioned above [13,59,60,61,72], the authors calculated the total time based on the experimental times of the RPA/EPA/LAMP and Cas12a/Cas13a reactions. We believe it to be illogical. The reason is that the viral DNA needs to be extracted prior to the EPA, LAMP, and RPA, which will also take some time. Meanwhile, the preparation of crRNA primers is also taking some time. Therefore, as seen in Figure 1, a scientific time should incorporate three stages.

### 3.6. Sensitivity

In ERA/LAMP/RPA-CRISPR-Cas12a/13a detection, the minimum and maximum LOD are 1 copy/µL and 10^3^ copies (Table 1), respectively. Wang et al. developed an RPA assay to detect PCV3 using a minimum of 23 copies [49], and a TaqMan-based method with a sensitivity as low as 15 copies/µL was created by Yuan et al., using primers and probe screening [73]. However, the LOD of G-quadruplex is only 10^3^ copies [61], which is quite high and requires more optimization to raise the LOD. In any case, the ERA/LAMP/RPA-CRISPR-Cas12a/Cas13a system coupled with FAM-BHQ1/BIO as reported in one study demonstrated its great sensitivity in comparison to earlier techniques and its capacity in PCV detection with as little as 1 copy/µL [13].

### 3.7. Specificity

When combined with the isothermal amplification of ERA/LAMP/RPA, the CRISPR-Cas12a/Cas13a system demonstrated high specificity for PCV detection. In real samples, mixed swine virus infections are highly prevalent, particularly when co-infection with PCVs occurs. Using the genomic DNA or RNA of a variety of porcine pathogenic viruses as templates, all the discussed studies (Table 1) confirmed the specificity of the ERA/LAMP/RPA-CRISPR-Cas12a/Cas13a assay. These viruses included CSFV, PCV2, PCV1, PCV3, PPV, PEDV, and PRRSV [59], PCV2, PCV4, PEDV, PRRSV, PRV, and CSFV [13], PCV2, PCV3, PRV, PPV, PRRSV, JEV, and CSFV [72], and PCV3, PRV, CSFV, ASFV, and PCV2 [60]. The results showed that fluorescence or brightness was only detected when PCVs were present, indicating the excellent specificity of this approach.

### 3.8. Reliability

With the exception of the study carried out by Wang et al. [61], the reliability of ERA/LAMP/RPA-CRISPR-Cas12a/Cas13a, which was established in all studies (Table 1) referred to here, was further confirmed by comparing it with the frequently used Taqman-based qPCR in detecting 30 clinical samples from 30 diseased pigs collected across different areas [59], 30 clinical samples [13], 15 clinical samples [72], and 20 clinical samples [60]. The comparison revealed that the reliability of ERA/LAMP/RPA-CRISPR-Cas12a/Cas13a was comparable to that of the current gold standard of qPCR.

### 3.9. Basic Requirements for Operators

Firstly, the operators need to have a basic background in biology. The reason is that all the other procedures can be designed and executed ahead of time by specific technicians. However, the actual operator must extract the genome of the virus sample on their own [13,59,60,61,72], which is the most crucial step since it lays a foundation for all subsequent experiments. Therefore, well-trained personnel ought to be present. Of course, the designation of ERA/LAMP/RPA primers and crRNAs is also a highly specialized task. Fortunately, you can use online web services to complete the primer designation. In addition, all the experiments in stages two and three are simple (Figure 1). According to the experimenter’s guide, the actual operator only needed to combine all the ingredients and begin the reaction in a temperature-control apparatus, such as a water bath or metal bath.

## 4. Conclusions

These ERA/LAMP/RPA-CRISPR-Cas12a/Cas13a-based PCV detection methods could be classified into three stages (Figure 1) after a series of optimizations of their reaction conditions. These stages may be carried out using inexpensive, constant-temperature equipment, and the results can be seen immediately by the naked eye. The lowest LODs of ERA/LAMP/RPA-CRISPR-Cas12a/Cas13a-based PCV detection methods are 1 copy/µL with no cross-reaction with major porcine DNA or RNA viruses such as PRV, PPV, PRRSV, JEV, and CSFV. And a 100% coincidence rate with the qPCR method in the assessment of actual samples demonstrates the fine sensitivity, specificity, and reliability of these ERA/LAMP/RPA-CRISPR-Cas12a/Cas13a-based PCV detection methods. Thus, this new technique facilitates the prevention of these pathogens in the field by enabling on-site, visual, extremely sensitive, and specific detection of PCVs [59]. Naturally, we advise using commercial kits for virus genome extraction and isothermal amplification. Commercial Cas12a and Cas13a proteins were also highly advised. Meanwhile, the overall cost times exceed the times that the authors have indicated. Therefore, the actual operators need to allow adequate time for PCVs to be detected by ERA/LAMP/RPA-CRISPR-Cas12a/Cas13a. In addition to Cas12a and Cas13a, other Cas proteins, such as Cas12b [74] and Cas13d [75], have also been employed in the detection of certain other types of viruses. Consequently, a growing amount of Cas proteins may be employed in PCV detection. Additionally, prior to detection, all the CRISPR-based PCV detection methods demonstrated here required genome extraction and nucleic acid amplification. Conversely, a novel cascade CRISPR-Dx system allows for detection without the need for amplified nucleic acids [76]. Researchers may investigate a growing variety of time- and labor-saving PCV detection methods based on CRISPR and isothermal amplification.

## Figures and Tables

**Table 1:** Experimental conditions for PCV detection using CRIPSR-Cas12a/13a and isothermal amplification.

The Kit Used for Viral DNA Extraction and Costed Time	Isothermal Amplification Method and Number of Primers	Cas Protein	The Number of Synthesized crRNA	Isothermal Amplification Kit and Time	Nucleic Acid Detection Temperature and Costed Time	Target	Detection Probe	LOD	Total Time		Reference
									By Calculation	By Authors	
TIANamp viral DNA/RNA kit (TIANGEN, Beijing, China), 60 min	LAMP, 6 primers	LbCas12a protein was expressed and purified by the authors	3 pairs	10 × LAMP Master Mix, 30 min at 63 °C	30 min at 37 °C	*rep* gene of PCV2	FAM-BHQ1-labeled ssDNA reporter	1 copy/µL	120 min	Within 60 min	[59]
Not reported	RPA, 1 pair	LbCas12a (M0653T, Beijing, NEB)	1 pair	TwistAmp^®^ Basic RPA kit (Maidenhead, UK), 20 min at 37 °C	45 min at 37 °C	*cap* gene of PCV2	G-quadruplex	>8.3 copies/µL	65 min+	Not reported	[61]
Not appliable	LAMP, 4	LbCas12a (New England Biolabs, NEB)	5	4 × LAMP Master Mix (Tianjingsha, Lianyungang, China), 60 min at 65 °C	50 min at 37 °C	ORF2 gene of PCV2	5′- FAM- TTATT-BHQ-1- 3′	1 copy/µL	110 min+	Not reported	[60]
TIANamp viral DNA/RNA kit (TIANGEN, Beijing, China), 60 min	ERA, 1 pair	EnGenVR Lba Cas12a (Cpf1; New England Biolabs, Ipswich, MA, USA)	3 pairs	A basic ERA nucleic acid amplification kit (GenDx Biotechnology, Suzhou, China), ~37 °C–40 °C for 20 min	20–30 min at 37 °C	*rep* gene of PCV3	FAM-N6-BHQ1	7 copies/µL	100–110 min	60 min	[13]
TIANamp Virus DNA/RNA Kit (TIANGEN, Beijing, China), 60 min	RPA, 5 pairs	LwaCas13a (Magiltd, China)	5 pairs	RPA kit (DNA-LS01, LeSunBio, Wuxi, China), 40 min at 37 °C	40 min at 37 °C	*cap* gene of PCV4	FAM-N6-BIO and LFD	1 to 10 copies/µL	140 min	90 min	[72]

Note: Enzymatic recombinase amplification (ERA); loop-mediated isothermal amplification (LAMP); recombinase polymerase amplification (RPA); lateral flow dipstick (LFD). +: more than the indicated time.

## Data Availability

Our study did not report any data.
